# Multifactorial Risk Stratification in Patients with Heart Failure, Chronic Kidney Disease, and Atrial Fibrillation: A Comprehensive Analysis

**DOI:** 10.3390/life15050786

**Published:** 2025-05-14

**Authors:** Mihai Sorin Iacob, Nilima Rajpal Kundnani, Abhinav Sharma, Vlad Meche, Paul Ciobotaru, Ovidiu Bedreag, Dorel Sandesc, Simona Ruxanda Dragan, Marius Papurica, Livia Claudia Stanga

**Affiliations:** 1 Doctoral School, “Victor Babes” University of Medicine and Pharmacy, 3000041 Timisoara, Romania; 2 University Clinic of Internal Medicine and Ambulatory Care, Prevention and Cardiovascular Recovery, Department VI—Cardiology, “Victor Babes” University of Medicine and Pharmacy, 3000041 Timisoara, Romania; 3 Research Centre of Timisoara Institute of Cardiovascular Diseases, “Victor Babes” University of Medicine and Pharmacy, 3000041 Timisoara, Romania; 4 University Clinic of Anaesthesia and Intensive Care, Department X Surgery II, “Victor Babes” University of Medicine and Pharmacy, 3000041 Timisoara, Romania; 5 Department XIV—Microbiology, “Victor Babes” University of Medicine and Pharmacy, 3000041 Timisoara, Romania

**Keywords:** hypertension, heart failure, chronic kidney disease, atrial fibrillation

## Abstract

**Background:** Heart failure (HF), chronic kidney disease (CKD), and atrial fibrillation (AF) frequently coexist, forming a high-risk triad that amplifies morbidity and mortality through shared pathophysiological mechanisms such as neurohormonal activation, fluid overload, and inflammation. Current risk stratification tools, including CHA_2_DS_2_-VASc and HAS-BLED, inadequately capture the complexity of these multimorbid patients. This study aims to explore the influence of comorbidities, hypertension severity, anticoagulation strategy, and risk scores on hospitalization outcomes in patients with coexisting HF, CKD, and AF. **Materials and Methods:** A retrospective case study was conducted on 174 hospitalized patients with HF, CKD, and AF. Clinical data included hypertension grade, HF phenotype (HFpEF vs. HFrEF), NYHA classification, renal function (KDIGO stage), stroke and bleeding risk scores (CHA_2_DS_2_-VASc: congestive heart failure, hypertension, age ≥ 75, diabetes, and stroke/TIA; HAS-BLED: hypertension, abnormal renal/liver function, stroke, bleeding, labile INR, elderly, and drugs/alcohol), comorbidities (neurological, psychiatric, COPD, and diabetes), anticoagulation type (DOACs vs. VKAs), and length of hospital stay. Statistical analysis included Spearman correlation, independent t-tests, and multivariate regression to evaluate associations between variables and clinical outcomes. **Results:** Most patients were elderly (mean age 75 ± 12), with advanced CKD (stage 3b) and systolic HF (77% HFrEF). Mean CHA_2_DS_2_-VASc was 5.67, HAS-BLED was 4.40, and ATRIA was 4.74, indicating high stroke and bleeding risk. Anticoagulation was predominantly via DOACs (69.5%). Hypertension severity did not significantly correlate with NYHA class (ρ = −0.14, *p* = 0.068). Neurological, psychiatric, and metabolic comorbidities showed no significant associations with HF severity. COPD and diabetes correlated inversely with CHA_2_DS_2_-VASc scores (ρ = −0.83, *p* = 0.014). No significant differences were observed in hospital stay between HF phenotypes or prior stroke history. In-hospital mortality was low (2.3%). **Conclusions:** Traditional risk scores do not fully capture the complexity of multimorbid patients. Metabolic comorbidities showed an inverse correlation with stroke risk scores, and no significant correlation was observed between hypertension severity and HF symptom burden. Hypertension and common comorbidities did not correlate with HF symptom burden, and metabolic diseases may paradoxically associate with lower stroke risk scores. These findings highlight the need for improved multimodal risk assessment strategies that consider the heterogeneity of multimorbid populations. Personalized, integrated approaches are essential to optimize anticoagulation, reduce hospitalization, and improve prognosis.

## 1. Introduction

Heart failure (HF), chronic kidney disease (CKD), and atrial fibrillation (AF) often coexist and synergistically worsen patient outcomes through shared mechanisms such as neurohormonal activation, inflammation, and hemodynamic stress. This triad is increasingly encountered in elderly, multimorbid populations and is associated with high hospitalization rates and mortality [1].

Recent studies emphasize the limitations of traditional risk scores—such as CHA_2_DS_2_-VASc for stroke and HAS-BLED for bleeding—in accurately stratifying risk in complex patients with multiple comorbidities [2]. Additionally, renal dysfunction is now recognized as a key modifier of cardiovascular risk, yet it remains under-represented in standard scoring systems [3,4].

There are limited data on how hypertension severity and non-cardiac comorbidities (neurological, psychiatric, pulmonary, and metabolic) influence heart failure symptom burden and risk stratification in patients with coexisting HF, CKD, and AF [5]. Previous models often consider each disease in isolation, which may overlook critical interdependencies [6,7].

Understanding the integrated risk landscape of these patients is vital for improving outcomes. Clarifying whether common comorbidities impact symptom severity or risk scores can refine treatment decisions, particularly around anticoagulation and heart failure management.

We hypothesized that the severity of hypertension and non-cardiac comorbidities would not significantly influence HF symptom burden (NYHA class) or traditional AF risk scores in this population. We also expected a disconnect between metabolic disease burden and stroke risk prediction tools.

The objective of this study was to assess the impact of hypertension, renal dysfunction, and comorbidities on HF symptom burden, stroke and bleeding risk scores, and hospitalization outcomes in patients with coexisting HF, CKD, and AF.

## 2. Pathophysiological Interplay of Heart Failure, Chronic Kidney Disease, Atrial Fibrillation, and Hypertension

The coexistence of heart failure (HF), chronic kidney disease (CKD), atrial fibrillation (AF), and hypertension represents a complex clinical scenario where each condition can exacerbate the others, leading to a vicious cycle of worsening health outcomes.

### 2.1. Shared Pathophysiological Mechanisms

The heart and kidneys are intricately linked through hemodynamic, neurohormonal, and inflammatory pathways. The concept of cardiorenal syndrome illustrates this bidirectional relationship, where dysfunction in one organ can precipitate dysfunction in the other. In HF, reduced cardiac output can lead to decreased renal perfusion, activating the renin–angiotensin–aldosterone system (RAAS) and sympathetic nervous system (SNS) [8]. This activation promotes sodium and water retention, increasing blood volume and further burdening the failing heart [9]. Simultaneously, CKD can exacerbate HF through fluid overload and the accumulation of uremic toxins that impair myocardial function [10].

### 2.2. Impact of Hypertension on the Triad of HF, CKD, and AF

Hypertension is a common antecedent to HF, CKD, and AF. Elevated blood pressure induces left ventricular hypertrophy, increasing myocardial oxygen demand and leading to HF over time. In the kidneys, hypertension damages glomeruli, reducing filtration capacity and advancing CKD. The structural and electrical remodeling of the atria due to hypertension predisposes individuals to AF [11]. The presence of AF can further deteriorate cardiac output and renal perfusion, creating a feedback loop that perpetuates disease progression [12].

### 2.3. Hemodynamic and Metabolic Dysregulation

The interplay among these conditions leads to significant hemodynamic and metabolic disturbances. Volume overload from HF and CKD results in increased central venous pressure, which can impair renal function—a phenomenon known as congestive nephropathy [13]. Metabolic derangements, such as electrolyte imbalances (e.g., hyperkalemia and hypocalcemia), are common and can exacerbate arrhythmias like AF [14]. Moreover, insulin resistance and dyslipidemia, often present in these patients, contribute to endothelial dysfunction and a pro-inflammatory state, further complicating the clinical picture [15].

## 3. Risk Stratification in Patients with HF, CKD, and AF

Effective risk stratification is crucial for managing patients with coexisting HF, CKD, and AF, as it guides therapeutic decisions and prognostication.

### 3.1. Established Risk Scores for Clinical Assessment

Several risk scores have been developed to predict adverse outcomes in these patients. The CHA_2_DS_2_-VASc score assesses stroke risk in AF patients by considering factors like heart failure, hypertension, age, diabetes, and prior stroke [16]. The HAS-BLED score evaluates bleeding risk in patients on anticoagulation therapy [17]. For HF, the New York Heart Association (NYHA) classification categorizes patients based on symptom severity [18]. In CKD, the Kidney Disease: Improving Global Outcomes (KDIGO) guidelines provide a framework for staging kidney disease and assessing progression risk [19].

### 3.2. Limitations of Current Risk Scores in Multimorbid Patients

While these scores are valuable, they often consider each condition in isolation, which may not accurately reflect the compounded risk in patients with multiple comorbidities. For instance, the CHA_2_DS_2_-VASc score may underestimate stroke risk in patients with both AF and CKD, as renal impairment independently increases thromboembolic risk [20]. Similarly, traditional HF risk models may not account for the impact of concurrent CKD or AF on outcomes [21]. This limitation underscores the need for integrated risk assessment tools that encompass the multifaceted nature of these overlapping conditions.

### 3.3. Emerging Approaches to Risk Stratification

Advancements in machine learning and artificial intelligence offer the potential to develop more sophisticated risk prediction models that integrate a multitude of variables, including biomarkers, genetic data, and real-time clinical parameters [22]. Biomarkers such as B-type natriuretic peptide (BNP), high-sensitivity troponins, and cystatin C have shown promise in enhancing risk stratification by providing insights into cardiac stress, myocardial injury, and renal function, respectively [23]. These approaches aim to facilitate personalized medicine, allowing for tailored therapeutic strategies that address the unique risk profile of each patient.

## 4. The Role of Renal Function in Cardiovascular Risk Stratification

Renal function is a pivotal determinant of cardiovascular outcomes, particularly in patients with concomitant HF and AF.

### 4.1. CKD Progression and Cardiovascular Outcomes

CKD is a significant risk factor for cardiovascular diseases, including coronary artery disease, HF, and arrhythmias like AF. The decline in the glomerular filtration rate (GFR) is associated with increased arterial stiffness, left ventricular hypertrophy, and a pro-inflammatory milieu, all of which contribute to adverse cardiovascular events [7]. Moreover, CKD-related mineral bone disorders can lead to vascular calcification, further elevating cardiovascular risk [24].

### 4.2. Renal Biomarkers and Risk Prediction

Beyond traditional measures like serum creatinine and estimated GFR, novel biomarkers have emerged as valuable tools for risk assessment. For example, elevated levels of fibroblast growth factor-23 (FGF-23) and soluble urokinase plasminogen activator receptor (suPAR) have been linked to increased cardiovascular mortality in CKD patients [25,26]. These biomarkers reflect underlying pathophysiological processes such as phosphate metabolism dysregulation and systemic inflammation, offering additional prognostic information beyond conventional risk factors.

### 4.3. Challenges in Anticoagulation Decision Making in CKD

#### 4.3.1. Balancing Thromboembolic vs. Bleeding Risk in CKD Patients with AF

Chronic kidney disease (CKD) significantly complicates anticoagulation therapy in atrial fibrillation (AF) patients due to the delicate balance between thromboembolic and bleeding risks. CKD is an independent risk factor for both ischemic stroke and major bleeding, making treatment decisions particularly challenging. Impaired renal function is associated with increased platelet dysfunction, endothelial dysfunction, and hypercoagulability, predisposing patients to thromboembolic events. Simultaneously, CKD is linked to a higher risk of bleeding due to platelet abnormalities, uremic toxins, and altered metabolism of anticoagulants [27].

Patients with CKD and AF are at a heightened risk of cardioembolic stroke, particularly those with estimated glomerular filtration rates (eGFRs) < 60 mL/min/1.73 m^2^. Studies indicate that CKD is an independent predictor of stroke in AF patients, with worsening renal function correlating with increased stroke incidence. However, the presence of CKD also raises the bleeding risk, particularly in advanced stages (eGFR < 30 mL/min/1.73 m^2^), where uremic platelet dysfunction and vascular calcifications increase hemorrhagic complications [28].

Current guidelines recommend anticoagulation for AF patients with CHA_2_DS_2_-VASc scores ≥2 in men and ≥3 in women, but the decision becomes more nuanced in CKD [29]. The HAS-BLED score, commonly used to assess bleeding risk, may underestimate hemorrhagic complications in CKD patients. Instead, comprehensive risk stratification using biomarkers (e.g., cystatin C and albuminuria) and individualized clinical assessment is necessary [30].

A meta-analysis found that in non-dialysis CKD patients with AF, anticoagulation reduced stroke risk but at the cost of an increased bleeding tendency [31]. Therefore, clinicians must carefully weigh stroke prevention benefits against the potential for life-threatening bleeding, particularly in patients with advanced CKD.

#### 4.3.2. Use of Direct Oral Anticoagulants (DOACs) vs. Vitamin K Antagonists (VKAs)

The selection of an appropriate anticoagulant for CKD patients with AF is a crucial decision. Traditionally, vitamin K antagonists (VKAs), such as warfarin, were the only available option. However, direct oral anticoagulants (DOACs), including dabigatran, rivaroxaban, apixaban, and edoxaban, have emerged as attractive alternatives due to their predictable pharmacokinetics, fewer food and drug interactions, and lack of requirement for frequent monitoring.

VKAs remain widely used in advanced CKD (eGFR < 30 mL/min/1.73 m^2^), but they pose significant challenges. Warfarin use in CKD patients has been associated with a paradoxical increase in vascular calcification, possibly due to vitamin K depletion. Moreover, time in therapeutic range (TTR) is often suboptimal in CKD patients, leading to increased thromboembolic and hemorrhagic events [32].

DOACs have demonstrated favorable safety and efficacy profiles in patients with mild-to-moderate CKD (eGFR ≥ 30 mL/min/1.73 m^2^). A landmark study showed that DOACs were associated with lower rates of intracranial hemorrhage and major bleeding compared to warfarin. However, in patients with severe CKD (eGFR < 15 mL/min/1.73 m^2^) or those on dialysis, clinical trial data are limited, and warfarin remains the standard therapy [33].

Apixaban has shown promising results in patients with advanced CKD and dialysis, with observational studies suggesting lower bleeding risks compared to warfarin. Nonetheless, large-scale randomized controlled trials are needed to definitively determine the best anticoagulation strategy for this high-risk group.

## 5. Stroke Prevention in AF: Standard Guidelines

Atrial fibrillation (AF) significantly elevates the risk of stroke, necessitating effective anticoagulation strategies. Current guidelines recommend assessing stroke risk using the CHA_2_DS_2_-VASc score, which considers factors such as heart failure, hypertension, age, diabetes, and prior stroke. Patients with a score of 2 or higher are typically advised to commence anticoagulation therapy. Traditionally, vitamin K antagonists (VKAs), such as warfarin, were the mainstay of treatment. However, direct oral anticoagulants (DOACs), including dabigatran, rivaroxaban, apixaban, and edoxaban, have emerged as preferred options due to their predictable pharmacokinetics and reduced need for monitoring. Evidence from randomized controlled trials supports the efficacy and safety of DOACs in stroke prevention among AF patients [34].

### 5.1. Anticoagulation Challenges in HF and CKD

The coexistence of heart failure (HF) and chronic kidney disease (CKD) complicates anticoagulation management. CKD patients are at an increased risk of both thromboembolic events and bleeding complications. The pharmacokinetics of anticoagulants are altered in CKD, necessitating careful dose adjustments. For instance, while DOACs are preferred in CKD stages 1 to 3, their use in advanced CKD (stage 4 or higher) is controversial due to limited data and increased bleeding risk. VKAs, though effective, require meticulous monitoring to maintain therapeutic ranges, especially in CKD patients. A recent meta-analysis highlighted that DOACs have a superior safety and efficacy profile compared to VKAs in patients with non-dialysis-dependent CKD and AF, reducing stroke risk with fewer major bleeding events [29].

### 5.2. Personalized Approaches to Anticoagulation

Given the complexities in patients with HF, CKD, and AF, a personalized approach to anticoagulation is essential. Factors such as renal function, bleeding risk, patient preferences, and comorbidities should guide therapy choices. The SAMe-TT_2_R_2_ score can aid in predicting the quality of anticoagulation control with VKAs, assisting in decision making between VKAs and DOACs. Emerging anticoagulants, such as factor XI inhibitors like abelacimab, have shown promise in reducing bleeding risks while effectively preventing thromboembolic events. A recent study demonstrated that abelacimab significantly reduced bleeding episodes compared to rivaroxaban in AF patients. Additionally, non-pharmacological interventions, such as left atrial appendage occlusion, may be considered in patients with contraindications to long-term anticoagulation. Shared decision making, incorporating patient values and clinical judgment, remains paramount in optimizing anticoagulation therapy [35].

## 6. Impact of Hypertension on Clinical Outcomes in Multimorbid Patients

### 6.1. Blood Pressure Control in Patients with HF, CKD, and AF

Hypertension is a prevalent comorbidity in patients with HF, CKD, and AF, and its management is crucial for improving clinical outcomes. Optimal blood pressure (BP) targets in this population are subject to ongoing research and debate. Intensive BP control has been associated with reduced cardiovascular events; however, overly aggressive lowering may lead to adverse effects, particularly in patients with compromised renal function. A recent study indicated that intensive BP control did not significantly reduce alternative clinical outcomes, such as heart failure, myocardial infarction, stroke, or all-cause death, highlighting the need for individualized BP targets [36].

### 6.2. Role of Antihypertensive Agents in Disease Progression

Antihypertensive medications play a pivotal role in managing patients with HF, CKD, and AF. Renin–angiotensin–aldosterone system (RAAS) inhibitors, such as ACE inhibitors and angiotensin receptor blockers, are cornerstone therapies that provide benefits beyond BP reduction, including mitigating disease progression in both HF and CKD. Beta-blockers are essential in HF management and offer rate control in AF. However, their use in CKD is primarily limited to patients with heart failure, arrhythmia, or coronary heart disease, conditions often present in CKD patients. Mineralocorticoid receptor antagonists have shown benefits in reducing morbidity and mortality in HF patients and may confer renal protection. The selection of antihypertensive agents should consider the patient’s overall clinical profile, comorbidities, and potential drug interactions [37].

### 6.3. Individualized Hypertension Management Strategies

Management of hypertension in patients with HF, CKD, and AF requires a tailored approach. Resistant hypertension, defined as BP that remains above the target despite the use of three or more antihypertensive agents, is common in this cohort. Strategies to address resistant hypertension include optimizing diuretic therapy, considering mineralocorticoid receptor antagonists, and evaluating for secondary causes of hypertension. Emerging therapies, such as renal denervation, are under investigation for their potential to manage resistant hypertension, particularly in patients with CKD. Lifestyle modifications, including dietary sodium reduction, weight management, and physical activity, are foundational components of hypertension management and should be emphasized alongside pharmacological interventions.

## 7. Comorbidities and Their Influence on Risk Stratification

### 7.1. Common Comorbidities in HF, CKD, and AF Patients

Patients with heart failure (HF), chronic kidney disease (CKD), and atrial fibrillation (AF) often present with multiple comorbidities that significantly impact clinical outcomes and complicate management strategies. Common comorbid conditions include hypertension, diabetes mellitus, chronic obstructive pulmonary disease (COPD), anemia, and sleep apnea. The presence of these comorbidities contributes to increased morbidity and mortality in this patient population.

### 7.2. Polypharmacy and Drug–Drug Interactions

The coexistence of multiple chronic conditions often necessitates the use of multiple medications, leading to polypharmacy. While essential for managing complex diseases, polypharmacy increases the risk of drug–drug interactions (DDIs), adverse drug events, and medication non-adherence. A study highlighted that polypharmacy and severe potential DDIs are very common in older adults with cardiovascular diseases, emphasizing the need for vigilant medication review to prevent adverse outcomes. Another study demonstrated that multimorbidity and polypharmacy are common after acute coronary syndromes, with drug interactions linked to clinical events [38,39].

### 7.3. Multidisciplinary Management Approaches

Given the complexity of managing patients with HF, CKD, AF, and their comorbidities, a multidisciplinary approach is essential. This involves collaboration among cardiologists, nephrologists, primary care physicians, pharmacists, and other healthcare professionals to develop individualized care plans. Such coordinated care has been associated with improved outcomes, including reduced hospital readmissions and enhanced quality of life. A review emphasized the importance of incorporating multimorbidity into clinical practice guidelines to optimize care for patients with cardiovascular diseases [40].

## 8. Clinical Outcomes and Future Directions

### 8.1. Major Adverse Cardiovascular Events (MACE) and Mortality Trends

The interplay of HF, CKD, and AF significantly elevates the risk of major adverse cardiovascular events (MACEs), including myocardial infarction, stroke, and cardiovascular death. Studies have shown that patients with these overlapping conditions have higher mortality rates compared to those with a single condition. For instance, the presence of CKD in patients with AF has been associated with a higher incidence of stroke and mortality.

### 8.2. Gaps in Current Research and Future Perspectives

Despite advancements in understanding the interrelationship between HF, CKD, and AF, several gaps remain. There is a need for large-scale, randomized controlled trials focusing on this multimorbid population to establish evidence-based management strategies. Additionally, research into the development of integrated risk assessment tools that consider the cumulative effect of these conditions is warranted. The integration of artificial intelligence and machine learning in predicting clinical outcomes and personalizing treatment plans represents a promising avenue for future research.

Additionally, accumulating evidence highlights the pleiotropic benefits of heart failure therapies beyond their traditional hemodynamic effects. Recent studies [41] have shown that medications such as SGLT2 inhibitors and angiotensin receptor–neprilysin inhibitors confer anti-inflammatory, antifibrotic, and renal-protective effects, which may be particularly advantageous in patients with HF, CKD, and AF. These pleiotropic mechanisms could further support the rationale for their broader application in multimorbid populations.

### 8.3. Personalized Medicine in HF, CKD, and AF

The heterogeneity among patients with HF, CKD, and AF necessitates a personalized approach to treatment. This involves tailoring therapies based on individual risk profiles, genetic predispositions, and patient preferences. Advancements in genomics and biomarker discovery are paving the way for precision medicine, enabling clinicians to predict disease progression and response to therapy more accurately. Implementing personalized medicine has the potential to improve clinical outcomes and enhance the quality of life for patients with these complex conditions.

## 9. Subjects and Methods

### 9.1. Study Design and Population

This retrospective observational study involved 174 hospitalized patients diagnosed with HF, CKD, and AF. The dataset included the following:

- Demographics: age, gender, and comorbidity burden.
- Sample size and power considerations.

No a priori sample size calculation or power analysis was performed prior to data collection, as this was a retrospective observational study. The post hoc analysis revealed that the study had approximately 65% power to detect a small-to-moderate correlation (ρ = 0.14) between hypertension severity and NYHA class at a significance level of 0.05, suggesting that non-significant findings may partly reflect limited statistical power. The analysis included the following:

- Clinical parameters: HF phenotypes (HFpEF vs. HFrEF), NYHA classification, renal function (eGFR), and hypertension severity.
- Comorbidities: presence of neurological, psychiatric, pulmonary (COPD, chronic obstructive pulmonary disease), and metabolic (diabetes) disorders.
- AF risk scores: CHA_2_DS_2_-VASc, HAS-BLED, and ATRIA.
- Anticoagulation therapy: whether patients were receiving DOACs (direct oral anticoagulants) or VKAs (vitamin K antagonists).
- Hospitalization outcomes: length of stay and in-hospital mortality.

Recruitment: This retrospective study included 174 patients diagnosed with heart failure (HF), chronic kidney disease (CKD), and atrial fibrillation (AF), admitted between January 2022 and December 2023 at the Municipal Hospital Clinic of Emergency Medicine, Timisoara, Romania. Patients were selected from electronic medical records. A total of 200 records were screened, of which 174 met the inclusion criteria.

The inclusion criteria were confirmed diagnosis of HF (via echocardiography), CKD stage ≥ 2 (Kidney Disease: Improving Global Outcomes (KDIGO) classification), and documented AF on ECG or Holter monitoring.

The exclusion criteria were patients with active malignancy (n = 10), incomplete records (n = 8), or other major arrhythmias (n = 8).

### 9.2. Statistical Analysis

The purpose of this study is to analyze the following:

1. The correlation between renal function (eGFR) and HF severity (NYHA classification).
2. The association between thromboembolic and bleeding risk scores (CHADS-VASC, HAS-BLED, and ATRIA) and in-hospital mortality.
3. The differences in hospital stay duration between HFpEF and HFrEF patients.
4. The impact of anticoagulation (direct oral anticoagulants [DOACs] vs. vitamin K antagonists [VKAs]) on bleeding risk (HAS-BLED score).
5. The effect of previous stroke history on hospital stay duration.
6. The relationship between the number of comorbidities and NYHA classification.

- Spearman correlation was used to assess the relationship between the following:
  o HTA severity and HF severity (NYHA classification).
  o Neurological, psychiatric, and metabolic comorbidities and AF risk scores (CHA_2_DS_2_-VASc, HAS-BLED, and ATRIA).
- Independent t-tests compared the following:
  o CHA_2_DS_2_-VASc, HAS-BLED, and ATRIA scores between survivors and non-survivors.
  o Hospital stay duration between HFpEF and HFrEF patients.
  o HAS-BLED scores between DOAC and VKA users.
  o Hospital stay duration in patients with and without prior stroke.
- Multivariate regression analysis was performed to identify independent predictors of high-risk scores for stroke and bleeding.

A *p*-value <0.05 was considered statistically significant.

- The multivariate regression model included the following variables: hypertension severity, renal function (eGFR), the presence of diabetes, COPD, neurological disorders, and type of anticoagulation (DOACs vs. VKAs). Multicollinearity was assessed using the variance inflation factor (VIF), and all included variables had a VIF < 2, indicating low multicollinearity. The model fit was evaluated using the coefficient of determination (R^2^), which was 0.28, indicating that approximately 28% of the variance in the risk scores could be explained by the included variables.

## 10. Results

- Demographics: Almost half of the patients (48%) had multiple hospitalizations, indicating a population with advanced chronic diseases.

**Figure 1:**
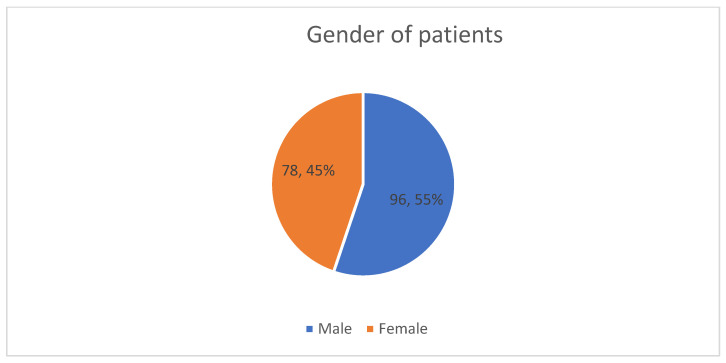
Distribution of genders among patients.

Distribution among genders was balanced (55% male and 45% female) (Figure 1).

The average length of hospital stay was approximately 3.4 (±2) days, consistent with the overall cohort data. A subgroup of patients with multiple comorbidities had longer hospitalizations (mean 10 ± 7.3 days).

The distribution of patients between urban and rural areas was relatively balanced (52% vs. 48%), with a slight predominance of those from rural areas.

**Figure 2:**
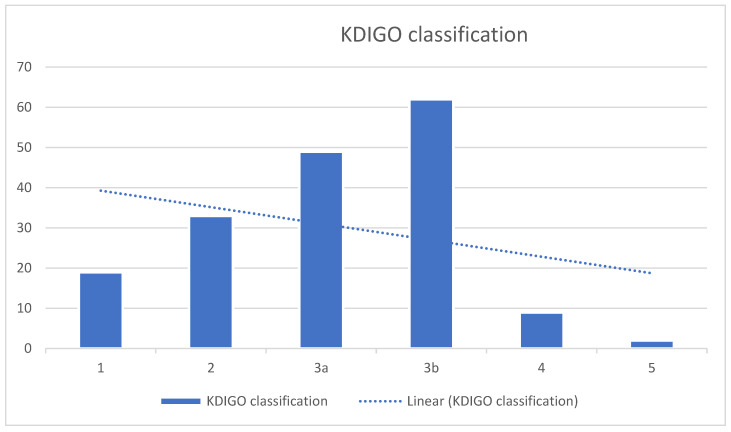
Distribution of kidney function stages based on the KDIGO classification among patients.

- Clinical parameters:

KDIGO classification: Most patients were in stage 3b (62 cases), indicating moderate–severe renal dysfunction (Figure 2).

**Figure 3:**
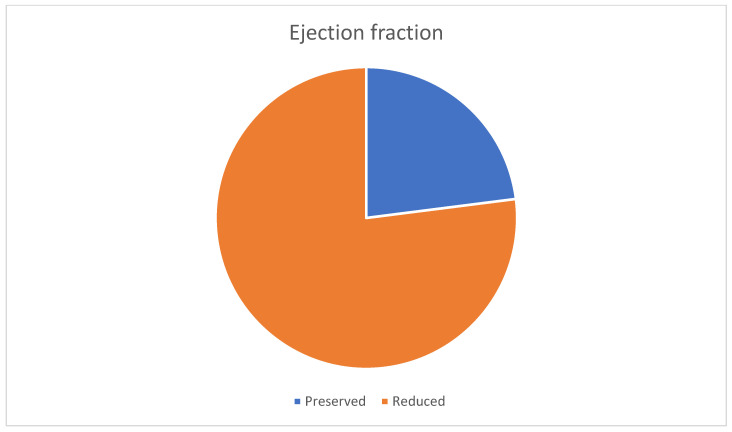
Distribution of ejection fraction among patients.

A total of 23% of the patients had a preserved ejection fraction, and 77% had a low ejection fraction, suggesting a predominance of heart failure with systolic dysfunction (Figure 3).

**Figure 4:**
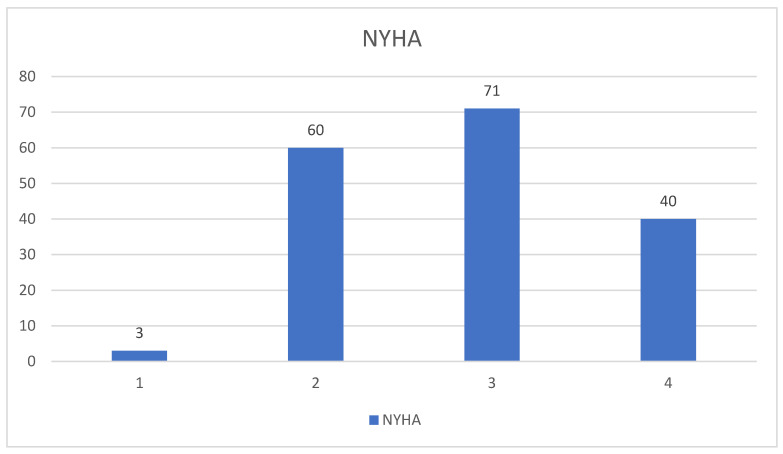
Distribution of NYHA classification of heart failure among patients.

NYHA classification: Most patients were NYHA II (34%) and NYHA III (40%), indicating moderate–severe symptomatic heart failure (Figure 4).

Very few patients were in NYHA I (asymptomatic) (three patients).

**Figure 5:**
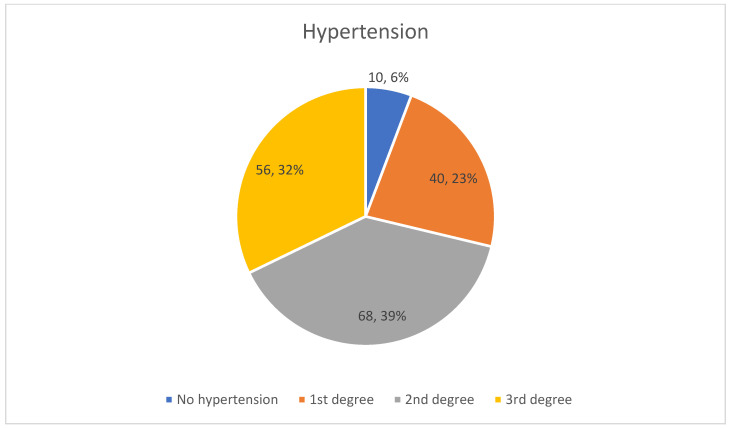
Distribution of hypertension among patients.

Hypertension was distributed among patients as follows: 10 patients (6%) had no hypertension, 40 patients (23%) had first-degree hypertension, 68 (39%) had second-degree hypertension, and 56 (32%) had third-degree hypertension. This shows that there is a strong correlation between the combination of AF-CKD-HF and hypertension (Figure 5).

**Figure 6:**
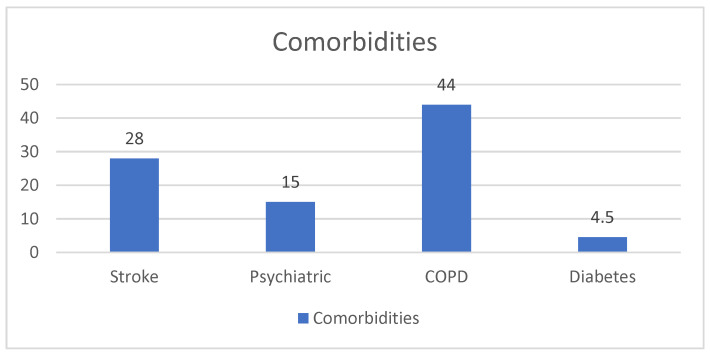
Comorbidities measured among patients.

- Comorbidities:

A total of 28 patients (16%) had suffered at least a stroke, and 15 patients (8, 6%) had at least one psychiatric disorder (dementia, bipolar disorder, schizophrenia, and Alzheimer’s disease). In addition, 44 patients (25%) had COPD, and 50 (28, 7%) were diabetic (Figure 6).

- Anticoagulation therapy: A total of 69, 5% (121) of patients were on DOACs, while 30, 4% (53) of patients were on VKAs.
- AF risk scores:

The mean CHADS-VASC score was 5.67 (±1.63), indicating a high risk of stroke in patients with atrial fibrillation.

Regarding the HAS-BLED score, the mean was 4.40 (±0.95), suggesting an increased risk of bleeding in patients on anticoagulant therapy.

Concerning the ATRIA score, the mean was 4.74 (±2.54), confirming a moderate–high risk of thromboembolic events.

- Hospitalization outcomes:

The average age of patients was approximately 75 (±12) years, suggesting an elderly population, typical of heart failure and chronic kidney disease.

The average length of stay in hospital was 3, 4 (±2) days.

In-hospital mortality was 2.3% (4 patients).

In summary, the baseline clinical characteristics of the patients by gender can be viewed in Table 1.

**Statistical Analysis**

### 10.1. Hypertension Severity and HF Symptom Burden (NYHA Classification)

**Spearman’s rho = −0.1388, and *p* = 0.0677 (weak negative correlation).**

Hypertension severity did not significantly correlate with HF symptom burden (NYHA class), suggesting that other factors (e.g., myocardial function, volume status, and renal function) may play a more dominant role in HF symptoms than blood pressure control alone.

### 10.2. Neurological Conditions vs. NYHA Score

**Spearman’s rho = −0.0417 (*p* = 0.5850).**

No significant correlation was found between neurological comorbidities (cortical atrophy, lacunarism, cognitive decline, and Parkinson’s disease) and NYHA classification.

### 10.3. Psychiatric Conditions vs. NYHA Score

**Spearman’s rho = −0.0143 (*p* = 0.8517),**

No meaningful relationship was found between psychiatric disorders (bipolar disorder, anxiety, depression, epilepsy, and dementia) and HF severity.

### 10.4. Other Comorbidities (COPD and Diabetes) vs. NYHA Score

**Spearman’s rho = −0.0721 (*p* = 0.3443).**

No significant correlation was found between COPD and diabetes and NYHA classification.

**The interpretation of these results is as follows:**

- Hypertension: there is a small negative association, which may indicate that hypertension severity is not a primary driver of symptomatic HF severity (NYHA).
- Neurological, psychiatric, and metabolic comorbidities do not seem to strongly influence HF symptom burden (NYHA class).
- This suggests that other factors (e.g., cardiac output, ejection fraction, and renal function) might play a larger role in determining functional limitation in HF patients.

### 10.5. Comorbidities and AF Risk Scores

#### 10.5.1. Neurological Conditions (e.g., Cortical Atrophy, Cognitive Decline, and Parkinson’s)

A weak positive trend was found with CHA_2_DS_2_-VASc scores (rho = 0.5868, *p* = 0.070).

No significant association with HAS-BLED or ATRIA scores was found.

#### 10.5.2. Psychiatric Conditions (e.g., Depression, Anxiety, and Bipolar Disorder)

No significant correlation with CHA_2_DS_2_-VASc, HAS-BLED, or ATRIA scores was found.

This suggests that psychiatric disorders do not significantly influence stroke or bleeding risk in patients with AF.

#### 10.5.3. COPD and Diabetes

A negative correlation was found with CHA_2_DS_2_-VASc scores (rho = −0.8358, *p* = 0.014, statistically significant).

No significant association with HAS-BLED or ATRIA scores was found.

This finding suggests that patients with COPD and diabetes may have lower CHA_2_DS_2_-VASc scores, possibly due to a lower prevalence of other vascular risk factors.

### 10.6. Hospitalization Outcomes

**Hospital Stay in HFpEF vs. HFrEF**

No significant difference in hospital stay duration (t = −1.02, *p* = 0.31) was found.

Both phenotypes of HF contribute to hospitalization burden, but the mechanisms differ.

**Stroke History and Hospital Stay**

No significant difference in hospital stay duration between patients with and without prior stroke was found (t = 1.21, *p* = 0.23).

Stroke history primarily impacts long-term outcomes rather than acute hospitalization duration.

## 11. Discussion

This retrospective study offers critical insights into the complex interplay between comorbidities and risk stratification scores in patients burdened with heart failure (HF), chronic kidney disease (CKD), and atrial fibrillation (AF)—a triad frequently encountered in clinical cardiology and internal medicine.

### 11.1. Comorbidities and AF Risk Scores

One of the most striking findings in our study was the inverse correlation between metabolic comorbidities (COPD and diabetes) and CHA_2_DS_2_-VASc scores (ρ = −0.8358, *p* = 0.014). Although counterintuitive, this suggests that certain high-risk patients may paradoxically be assigned lower stroke risk scores due to the under-representation of these comorbidities within traditional scoring criteria. This could be explained by age, gender distribution, or fewer overlapping risk factors (e.g., prior stroke and vascular disease) in the subgroup affected by COPD and diabetes. A similar trend was reported by Suhov et al. [42] in a 2025 Romanian cohort of AF patients with diabetes, where vascular complications did not consistently align with CHA_2_DS_2_-VASc scoring tiers. Internationally, a 2024 Spanish registry study [43] also highlighted that traditional scores may lack sensitivity in populations with multiple chronic conditions, reinforcing the need to re-evaluate how we weigh comorbidities in stroke prediction tools.

### 11.2. Hypertension Severity and HF Symptom Burden

The second key observation was that hypertension severity and comorbidity burden (neurological, psychiatric, COPD, and diabetes) did not significantly correlate with HF symptom severity, as measured by NYHA class. The small, non-significant correlation between HTA and NYHA (ρ = −0.1388, *p* = 0.0677) suggests that other pathophysiological drivers—such as left ventricular dysfunction, volume status, or renal impairment—play a more prominent role in functional limitation than blood pressure control alone. Similar findings were described in RELAX-AHF2 [44], where NYHA class aligned more closely with renal function and natriuretic peptide levels than with traditional comorbidities. Our results add support to the notion that in multimorbid patients, NYHA classification may inadequately reflect total disease burden, highlighting the limitations of symptom-based classification in complex cases.

### 11.3. Hospitalization Outcomes

The fact that there were no significant differences in hospitalization duration between HFpEF and HFrEF subgroups, as well as between patients with and without prior stroke, suggests that short-term hospital resource utilization may be similar across different HF phenotypes and stroke histories. This emphasizes that acute hospitalization drivers—such as decompensation triggers, renal dysfunction, and arrhythmia management—may play a more pivotal role than chronic disease history in length of stay. However, it is important to note that stroke history often influences long-term outcomes such as functional decline or recurrent hospitalizations, which were not captured in this study.

### 11.4. Implications for Clinical Practice

These findings highlight the importance of personalized risk stratification in patients with overlapping cardiovascular and renal comorbidities. While traditional risk scores remain useful, they may not fully account for atypical comorbidity profiles seen in real-world settings:

- Clinicians may overestimate stroke risk in patients with cognitive decline while underestimating it in those with metabolic disorders.
- Psychiatric conditions, despite being under-represented in scoring systems, could still affect clinical outcomes through adherence and follow-up challenges.

Recent developments in machine learning-based risk stratification offer promising avenues for improving outcome prediction in multimorbid populations. Models such as GARFIELD-AF [45] and the ABC [46] (age, biomarkers, and clinical history) score have demonstrated superior predictive performance compared to traditional risk calculators like CHA_2_DS_2_-VASc, particularly in heterogeneous cohorts. The integration of biomarker panels, imaging data, and real-time clinical parameters into algorithm-driven models could enhance personalized anticoagulation and heart failure management strategies in patients with overlapping HF, CKD, and AF.

The lack of differentiation in hospital stay also underscores the need for tailored inpatient strategies, regardless of HF phenotype, with an emphasis on early mobilization, volume management, and integrated care for coexisting conditions.

### 11.5. Limitations

Several limitations of this study must be acknowledged. First, the retrospective and single-center nature limits generalizability. The sample size, while adequate for exploratory analysis, may be underpowered to detect subtle associations. Additionally, disease severity and treatment nuances (e.g., diuretic dose and adherence to guideline-directed therapy) were not captured in full detail, potentially confounding the observed relationships. The inverse correlation between COPD/diabetes and stroke risk scores warrants cautious interpretation and validation in prospective studies.

### 11.6. Future Directions

Future research should aim to achieve the following:

- Validate these findings in larger, multicenter cohorts.
- Explore psychosocial and frailty markers as potential modifiers of stroke and bleeding risk.
- Investigate long-term outcomes, such as rehospitalization rates, mortality, and quality of life.
- Assess whether incorporating novel biomarkers or machine learning algorithms could enhance risk prediction in complex multimorbid populations.

## 12. Conclusions

This study highlights the complex interplay between hypertension, comorbidities, and atrial fibrillation risk scores. While hypertension severity does not directly impact NYHA classification, neurological conditions show a weak association with increased stroke risk. Psychiatric and metabolic comorbidities require further investigation to determine their true influence on AF risk scores and outcomes. A tailored, multimodal approach to patient care is essential for optimizing management strategies in this high-risk population.

## Figures and Tables

**Table 1:** Baseline characteristics of patients by gender.

Variable	Male (n = 96)	Female (n = 78)	Total (n = 174)
Mean age (years)	73 (±12.5)	76 (±13)	75 (±12)
Mean BMI (kg/m^2^)	27, 73	29, 53	28, 53
Hypertension (%)	94	93, 5	94, 25
Diabetes (%)	24	35	28, 7
Tobacco use (%)	72, 9	74, 35	77
Mean ejection fraction (%)	43, 65	42, 88	43, 31
Mean eGFR (mL/min/1.73 m^2^)	43, 96	42, 78	43, 44
Comorbidities (COPD, stroke, and psychiatric)	55, 2	44, 8	50

## Data Availability

All the data and materials will be made available on written request.
